# Structural genomics of bacterial drug targets: Application of a high-throughput pipeline to solve 58 protein structures from pathogenic and related bacteria

**DOI:** 10.1128/mra.00200-25

**Published:** 2025-05-20

**Authors:** Nicole L. Inniss, George Minasov, Changsoo Chang, Kemin Tan, Youngchang Kim, Natalia Maltseva, Peter Stogios, Ekaterina Filippova, Karolina Michalska, Jerzy Osipiuk, Lukasz Jaroszewki, Adam Godzik, Alexei Savchenko, Andrzej Joachimiak, Wayne F. Anderson, Karla J. F. Satchell, the Center for Structural Biology of Infectious Diseases Team members

**Affiliations:** 1 Department of Microbiology-Immunology, Northwestern University, Feinberg School of Medicine 547641, Chicago, Illinois, USA; 2 Center for Structural Biology of Infectious Diseases, Feinberg School of Medicine, Northwestern University 205058, Chicago, Illinois, USA; 3 Consortium for Advanced Science and Engineering, University of Chicago 2462, Chicago, Illinois, USA; 4 Structural Biology Center, X-ray Science Division, Argonne National Laboratory 1291, Lemont, Illinois, USA; 5 Biozone, Department of Chemical Engineering and Applied Chemistry, University of Toronto 7938, Toronto, Ontario, Canada; 6 Biosciences Division, University of California, Riverside, School of Medicine 378743, Riverside, California, USA; 7 Department of Microbiology, Immunology, and Infectious Diseases, University of Calgary 198998, Calgary, Alberta, Canada; 8 Department of Biochemistry and Molecular Genetics, Feinberg School of Medicine, Northwestern University 205058, Chicago, Illinois, USA; Indiana University, Bloomington, Bloomington, Indiana, USA

**Keywords:** X-ray, structure, antimicrobial agents, drug targets, bacteria, PDB

## Abstract

Antibiotic resistance remains a leading cause of severe infections worldwide. Small changes in protein sequence can impact antibiotic efficacy. Here, we report deposition of 58 X-ray crystal structures of bacterial proteins that are known targets for antibiotics, which expands knowledge of structural variation to support future antibiotic discovery or modifications.

## ANNOUNCEMENT

Antibiotic-resistant bacteria remain a global threat, with millions of deaths attributed to decreased drug efficacy (1, 2). Amino acid variation across different bacterial species can impact antimicrobials targeting essential biochemical pathways. To support antimicrobial discovery or chemical modification of current antibiotics, the Center for Structural Genomics of Infectious Diseases (now the Center for Structural Biology of Infectious Diseases [CSBID]) established a high-throughput (HTP) structural genomics pipeline to expand the diversity of structures available for proteins that are known drug targets. A list of proteins representing known antibiotic targets was curated using DrugBank (http://www.drugbank.ca/). The protein sequences were used as queries to identify homologs in bacterial species with genomic DNA available in the center repository. Proteins sharing at least 50% sequence identity across 75% of the protein sequence were selected. In total, 630 targets from 47 bacterial species entered the pipeline.

All targets were subjected to automated analyses supporting protein expression construct design. The genes encoding the selected proteins or protein domains were amplified by PCR using genomic DNA as a template. The PCR products were cloned into pMCSG53 (PSI:Biology-Materials Repository, http://psimr.asu.edu) according to published ligation-independent cloning procedures (3, 4). This vector introduced a protease-cleavable, N-terminal hexa-histidine purification tag. The clones were transformed into T7-polymerase expressing *Escherichia coli* strains and tested for expression and solubility. Soluble proteins were purified by nickel affinity chromatography according to published protocols (5, 6), and concentrated proteins were set up as 2-µL crystallization drops in 96-well plates using multiple screens. Resulting crystals were cryoprotected, cooled, and then screened for data collection at the Advanced Photon Source (APS) at Argonne National Laboratory.

**Fig 1:**
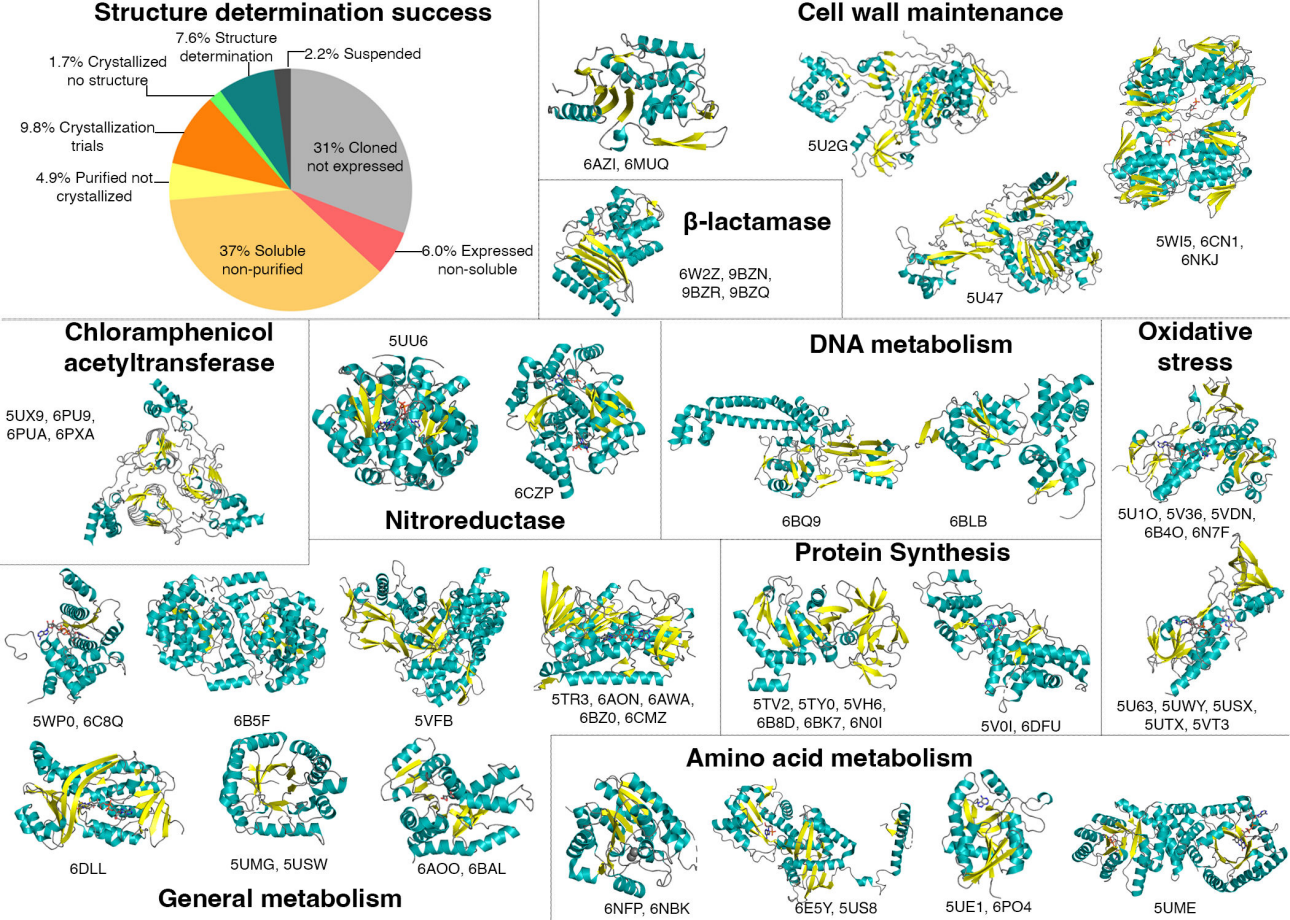
Percentage of approved bacterial drug targets at each stage in the structure determination pipeline and representative X-ray structures. The pie chart shows the overall success rate of proteins in the structure determination pipeline from a total of 630 targets. Work was completed between 2016 and 2024. Twenty-five representative structures are depicted as cartoons: β-sheets are colored yellow, α-helices are teal, and loops are gray. Associated crystal variants, complexes with ligands, and homologous structures are annotated below each image, totaling 58 structures solved. The proteins were sorted according to their known function in bacteria. Associated ligands and crystallographic details are described in Table 1.

In total, 24% of targets were purified, and 19% yielded protein preparations that entered HTP crystallization screens. Pipeline success rate from selection through structure determination was 7.6%. Forty-eight targets from 24 bacterial species produced high-quality crystals, yielding 58 structures (Fig. 1). The RCSB Protein Data Bank (PDB) deposition code, protein name, source DNA, and refinement statistics are listed in Table 1. Of the 58 structures determined, 55 are reported here for the first time, with three structures published previously (7, 8). Structures were derived from proteins involved in antibiotic modification, cell wall maintenance, oxidative stress, and metabolism.

**TABLE 1:** Summary of bacterial drug targets with structures deposited to Protein Data Bank^*a*^

PDB code	Csbid Ref #	Protein name	Organism	Resolution	Ligand^*b*^
6nbk	IDP07367	Arginase	*Bacillus cereus*	1.91 Å	–
6nfp	IDP07164	Arginase	*Bacillus subtilis*	1.70 Å	–
5us8	IDP07200	Argininosuccinate synthase	*Bordetella pertussis*	2.15 Å	Adenosine
6e5y	iDP07200	Argininosuccinate synthase	*Bordetella pertussis*	1.50 Å	AMP
6w2z	IDP07475	Beta-lactamase class A	*Bacillus subtilis*	1.50 Å	Avibactam
9bzn	IDP07519	Beta-lactamase class A	*Bordetella bronchiseptica*	1.05 Å	–
9bzq	IDP07519	Beta-lactamase class A	*Bordetella bronchiseptica*	1.47 Å	Avibactam
9bzr	IDP07519	Beta-lactamase class A	*Bordetella bronchiseptica*	1.40 Å	Clavulanate
6pua	IDP07511	Chloramphenicol acetyltransferase	*Vibrio cholerae*	2.00 Å	–
5ux9	IDP07301	Chloramphenicol acetyltransferase	*Vibrio fischeri*	2.70 Å	–
6pxa	IDP07301	Chloramphenicol acetyltransferase	*Vibrio fischeri*	1.82 Å	Taurocholic acid
6pu9	IDP07511	Chloramphenicol acetyltransferase	*Vibrio vulnificus*	1.70 Å	–
6b5f	IDP07570	CobT	*Yersinia enterocolitica*	1.95 Å	–
6azi	IDP07508	D-ala-D-ala-endopeptidase	*Enterobacter cloacae*	1.75 Å	–
6bz0	IDP07418	Dihydrolipoamide dehydrogenase	*Acinetobacter baumannii*	1.83 Å	FAD
6aon	IDP07182	Dihydrolipoamide dehydrogenase	*Bordetella pertussis*	1.72 Å	FAD
6cmz	IDP07673	Dihydrolipoamide dehydrogenase	*Burkholderia cenocepacia*	2.30 Å	FAD, NAD
6awa	IDP07540	Dihydrolipoamide dehydrogenase	*Pseudomonas aeruginos*a	1.83 Å	FAD, AMP
5tr3	IDP07540	Dihydrolipoamide dehydrogenase	*Pseudomonas putida*	2.50 Å	FAD
5umg	IDP07170	Dihydropteroate synthase	*Klebsiella pneumoniae*	2.60 Å	–
5usw	IDP07359	Dihydropteroate synthase	*Vibrio fischeri*	1.64 Å	–
6bq9	IDP07285	DNA Topoisomerase IV Subunit A	*Pseudomonas putida*	2.55 Å	–
–5vh6	IDP07716	Elongation factor G	*Bacillus subtilis*	2.61 Å	–
6bk7	IDP07555	Elongation factor G	*Enterococcus faecalis*	1.83 Å	–
6b8d	IDP07537	Elongation factor G	*Haemophilus influenzae*	1.78 Å	–
5ty0	IDP07381	Elongation factor G	*Legionella pneumophila*	2.22 Å	–
6n0i	IDP07336	Elongation factor G	*Pseudomonas putida*	2.60 Å	–
5tv2	IDP07581	Elongation factor G	*Vibrio vulnificus*	1.60 Å	–
6b4o	IDP07317	Glutathione reductase	*Enterococcus faecalis*	1.73 Å	FAD
5v36	IDP07311	Glutathione reductase	*Streptococcus mutans*	1.88 Å	FAD
6n7f	IDP07597	Glutathione reductase	*Streptococcus pyogenes*	1.90 Å	–
5u1o	IDP07224	Glutathione reductase	*Vibrio parahaemolyticus*	2.31 Å	FAD
5vdn	IDP07394	Glutathione reductase	*Yersinia pestis*	1.55 Å	FAD
6aoo	IDP07201	Malate dehydrogenase	*Haemophilus influenzae*	2.15 Å	–
6bal	IDP07201	Malate dehydrogenase	*Haemophilus influenzae*	2.10 Å	L-malate
5vfb	IDP07567	Malate synthase G	*Pseudomonas aeruginos*a	1.36 Å	Glycolytic acid
5ume	IDP07318	MetF	*Haemophilus influenzae*	2.70 Å	FAD
6po4	IDP07178	Methylthioadenosine/SAH nucleosidase	*Haemophilus influenzae*	2.10 Å	–
5ue1	IDP07462	Methylthioadenosine/SAH nucleosidase	*Vibrio fischeri*	1.14 Å	Adenine
6muq	IDP07205	Murein-DD-endopeptidase	*Yersinia enterocolitica*	1.67 Å	–
6c8q	IDP07348	NAD synthetase	*Enterococcus faecalis*	2.58 Å	NAD
5wp0	IDP07110	NAD synthetase	*Vibrio fischeri*	2.60 Å	–
5uu6	IDP07628	Nitroreductase A	*Vibrio parahaemolyticus*	1.95 Å	FMN
6czp	IDP07377	Nitroreductase A	*Vibrio vulnificus*	2.24 Å	FMN
6dll	IDP07306	p-Hydroxybenzoate Hydroxylase	*Pseudomonas putida*	2.20 Å	FAD
5u2g	IDP07344	Penicillin-binding protein 1A	*Haemophilus influenzae*	2.61 Å	–
5u47	IDP07211	Penicillin-binding protein 2X	*Streptococcus thermophilus*	1.95 Å	–
6blb	IDP07228	RuvB	*Pseudomonas aeruginosa*	1.88 Å	ADP
5u63	IDP07488	Thioredoxin reductase	*Haemophilus influenzae*	1.99 Å	–
5uwy	IDP07356	Thioredoxin reductase	*Streptococcus pyogenes*	2.72 Å	FAD
5utx	IDP07222	Thioredoxin reductase	*Vibrio vulnificus*	2.46 Å	–
5usx	IDP07222	Thioredoxin reductase	*Vibrio vulnificus*	2.60 Å	NADP, FAD
5vt3	IDP07222	Thioredoxin reductase	*Vibrio vulnificus*	1.98 Å	NADP, FAD
5v0i	IDP07325	Tryptophanyl-tRNA synthetase	*Escherichia coli*	1.90 Å	Tryptophan, AMP
6dfu	IDP07216	Tryptophanyl-tRNA synthetase	*Haemophilus influenzae*	2.05 Å	–
6cn1	IDP07215	UDP-GlcNAc 1-carboxyvinyltransferase	*Pseudomonas putida*	2.75 Å	UDP-GlcNAc
6nkj	IDP07236	UDP-GlcNAc 1-carboxyvinyltransferase	*Streptococcus pneumoniae*	1.30 Å	–
5wi5	IDP07236	UDP-GlcNAc 1-carboxyvinyltransferase	*Streptococcus pneumoniae*	2.00 Å	UDP-GlcNAc

^*a*^ Access link for Data quality and refinement statistics 10.5281/zenodo.15224721.

^*b*^ –, indicates no ligand.

Data collection and data quality information are available on the PDB. Structures of proteins grown in selenomethionine medium were solved by single-wavelength anomalous diffraction method, using the Automatic Structure Solution from HKL-3000 (9) and Auto-build package from PHENIX (10). Structures of native proteins were solved by molecular replacement in the CCP4 suite (11). Diffraction data were used for structure solution using either the structure of the closest sequence homolog in the PDB in PHASER or the target protein sequence using MORDA and MRBUMP. Structures were refined using REFMAC5 (12) or PHENIX and visually corrected in Coot (13). Water molecules were generated using ARP/wARP (14), and ligands were fit into electron density maps in Coot. Translation–Libration–Screw groups were generated by the TLSMD server (15), and corrections were applied during refinement finalization. Models were validated using MolProbity (16).

## Data Availability

The list of protein targets with solved structures is available at http://targets.csbid.org/targets organized as batch “set296” with the internal tracking number (IDP) listed in Table 1. The entire list of 630 protein targets including primer sequences and minor changes to protein purification can be found on the legacy database at csgid.org or by request from the Center for Structural Biology of Infectious Diseases. All coordinates for all final models and experimental data have been deposited to the Protein Data Bank (https://www.rcsb.org/), and can be found using PDB codes 5TR3, 5TV2, 5TY0, 5U1O, 5U2G, 5U47, 5U63, 5UE1, 5UME, 5UMG, 5US8, 5USW, 5USX, 5UTX, 5UU6, 5UWY, 5UX9, 5V0I, 5V36, 5VDN, 5VFB, 5VH6, 5VT3, 5WI5, 5WP0, 6AON, 6AOO, 6AWA, 6AZI, 6B4O, 6B5F, 6B8D, 6BAL, 6BK7, 6BLB, 6BQ9, 6BZ0, 6C8Q, 6CMZ, 6CN1, 6CZP, 6DFU, 6DLL, 6E5Y, 6MUQ, 6N0I, 6N7F, 6NBK, 6NFP, 6NKJ, 6PO4, 6PU9, 6PUA, 6PXA, 6W2Z, 9BZN, 9BZR, and 9BZQ.
